# Role of enhanced vector transmission of a new West Nile virus strain in an outbreak of equine disease in Australia in 2011

**DOI:** 10.1186/s13071-014-0586-3

**Published:** 2014-12-12

**Authors:** Andrew F van den Hurk, Sonja Hall-Mendelin, Cameron E Webb, Cindy S E Tan, Francesca D Frentiu, Natalie A Prow, Roy A Hall

**Affiliations:** Virology, Public and Environmental Health, Forensic and Scientific Services, Department of Health, Queensland Government, Brisbane, QLD Australia; Australian Infectious Disease Research Centre, School of Chemistry and Molecular Biosciences, University of Queensland, Brisbane, QLD Australia; Department of Medical Entomology, University of Sydney and Pathology West – ICPMR Westmead, Westmead, NSW Australia; Institute of Health and Biomedical Innovation and School of Biomedical Sciences, Queensland University of Technology, Kelvin Grove, QLD Australia

**Keywords:** Arbovirus, West Nile virus Kunjin strain, *Culex annulirostris*, Infection, Transmission, Australia

## Abstract

**Background:**

In 2011, a variant of West Nile virus Kunjin strain (WNV_KUN_) caused an unprecedented epidemic of neurological disease in horses in southeast Australia, resulting in almost 1,000 cases and a 9% fatality rate. We investigated whether increased fitness of the virus in the primary vector, *Culex annulirostris*, and another potential vector, *Culex australicus*, contributed to the widespread nature of the outbreak.

**Methods:**

Mosquitoes were exposed to infectious blood meals containing either the virus strain responsible for the outbreak, designated WNV_KUN2011_, or WNV_KUN2009_, a strain of low virulence that is typical of historical strains of this virus. WNV_KUN_ infection in mosquito samples was detected using a fixed cell culture enzyme immunoassay and a WNV_KUN_- specific monoclonal antibody. Probit analysis was used to determine mosquito susceptibility to infection. Infection, dissemination and transmission rates for selected days post-exposure were compared using Fisher’s exact test. Virus titers in bodies and saliva expectorates were compared using *t*-tests.

**Results:**

There were few significant differences between the two virus strains in the susceptibility of *Cx. annulirostris* to infection, the kinetics of virus replication and the ability of this mosquito species to transmit either strain. Both strains were transmitted by *Cx. annulirostris* for the first time on day 5 post-exposure. The highest transmission rates (proportion of mosquitoes with virus detected in saliva) observed were 68% for WNV_KUN2011_ on day 12 and 72% for WNV_KUN2009_ on day 14. On days 12 and 14 post-exposure, significantly more WNV_KUN2011_ than WNV_KUN2009_ was expectorated by infected mosquitoes. Infection, dissemination and transmission rates of the two strains were not significantly different in *Culex australicus*. However, transmission rates and the amount of virus expectorated were significantly lower in *Cx. australicus* than *Cx. annulirostris*.

**Conclusions:**

The higher amount of WNV_KUN2011_ expectorated by infected mosquitoes may be an indication that this virus strain is transmitted more efficiently by *Cx. annulirostris* compared to other WNV_KUN_ strains. Combined with other factors, such as a convergence of abundant mosquito and wading bird populations, and mammalian and avian feeding behaviour by *Cx. annulirostris*, this may have contributed to the scale of the 2011 equine epidemic.

## Background

West Nile virus (WNV) is a mosquito-borne flavivirus that historically was responsible for outbreaks of acute encephalitis in Africa, Europe, Russia and the Middle East [1], but is most notable for its emergence in the Americas [2]. The Kunjin strain of WNV (WNV_KUN_) is endemic in Australia, where it can cause a mild febrile illness and occasionally non-fatal encephalitis in humans and horses [3]. The virus is endemic in northern Australia and occasionally spreads into southern regions when heavy rainfall and flooding create an ideal environment for ardeid birds, the key amplifying hosts, and the proliferation of *Culex annulirostris*, the primary mosquito vector [4].

Between January and June 2011, a widespread outbreak of neurological disease attributed to arbovirus infection occurred amongst horses in southeastern Australia resulting in 982 reported equine cases, with an overall case fatality rate of 9% [5]. In addition to Ross River virus and Murray Valley encephalitis virus (MVEV), a WNV-like virus was isolated from mosquitoes and deceased horses, and was revealed antigenically and genotypically to be a strain of WNV_KUN_ [6]. This strain of WNV_KUN_, designated WNV_KUN2011_, was subsequently shown to be the primary cause of neurological disease in horses [5,6]. This was unexpected, because unlike the North American strain of WNV, which is highly pathogenic to horses, WNV_KUN_ is only rarely associated with equine disease [3,7]. Paradoxically, there were very few human clinical cases attributed to infection with WNV_KUN_ from epidemic foci during 2011 [6]. Furthermore, a cross-sectional serosurvey of 1,115 human serum specimens from a focus of WNV_KUN_ in Victoria found less than 0.3% were IgM positive, providing little evidence of recent exposure amongst the human population [8]. In contrast to North American WNV, which is highly pathogenic in a number of bird species [9,10], there was no increased mortality observed in birds during the 2011 outbreak [6].

Of note, other key epidemiological and biological features characterized this unique outbreak. La Niña-driven widespread rainfall and extensive flooding in southeastern Australia during the spring and summer of 2010–2011 [11] triggered an explosion of mosquito numbers, particularly in inland areas. Indeed, over 200,000 mosquitoes were collected during the 2010–2011 season in New South Wales, which was considerably higher than numbers collected in the preceding two years [12]. WNV_KUN_ was also reported in novel areas, such as east of the Great Dividing Range near the major coastal population centers of Sydney, Newcastle and Wollongong [5,6]. This included the first virus isolate from mosquitoes collected from the eastern seaboard [12].

A key factor that may have led to the 2011 outbreak was increased fitness of WNV_KUN2011_ in mosquitoes, similar to what has been suggested for emergent strains of WNV in North America [13,14]. To examine the mechanisms of its emergence, we report the characterization of the WNV_KUN2011_ strain in *Cx. annulirostris* and compare it to a recent strain of WNV_KUN_ that has not been associated with recognized pathogenicity in horses or other vertebrates. Infection characteristics of the two virus strains were also evaluated in another potential vector, *Culex australicus*. This species is an indigenous member of the *Culex pipiens* group, a complex which contains major WNV vectors in North America and Europe [15,16]. It has a wide distribution across southern Australia, south of 17°S [17], including the region affected by the epidemic.

## Methods

### Virus strains

The WNV_KUN2011_ was isolated from the brain of a deceased horse from Boorowa (34°28′S; 148°45′N), NSW. It had been passaged three times, once each in baby hamster kidney (BHK), African green monkey (Vero) and *Aedes albopictus* (C6/36) cells and the stock virus had a final titre of 10^8.9^ tissue culture infectious dose (TCID)_50_/mL. The strain to which it was compared (WNV_KUN2009_) was isolated from *Cx. annulirostris* collected from Kununurra (15°46′S; 128°44′N), Western Australia, in 2009. It had been passaged twice in C6/36 cells, once in porcine stable equine kidney (PSEK) cells before a final passage in C6/36 cells; the stock virus had a final titre of 10^8.7^ TCID_50_/mL.

### Mosquitoes

Adult *Cx. annulirostris* and *Cx. australicus* were collected from a number of sites around Hexham Swamp (32°50′S; 151°40′E), Fullerton Cove (32°50′S; 151°50′E) and Kooragang Island (32°52′S;151°45′E) near Newcastle, NSW, using encephalitis virus surveillance (EVS) light traps [18] baited with CO_2_ (approx. 1 kg dry ice). The mosquitoes were transported to the laboratory and placed in a 30 × 30 × 30 cm cage at 26°C, 75% humidity and 12:12 L:D. On the night following collection, mosquitoes were offered an anesthetized rat as a blood meal source. The use of animals was approved by the University of Sydney and Westmead Hospital Animal Ethics Committee (approval number 8001/04-10). Two days later, 200 mL of water was added to the cage for oviposition. After two days, egg rafts were removed, placed on moist filter paper in a Petri dish and forwarded by overnight courier to Forensic and Scientific Services, Department of Health, Brisbane.

Upon receipt, eggs were hatched in 2.5 L of ddH_2_O containing ~ 45 mg of brain-heart infusion powder. First and 2nd instar larvae were fed a 1:1 mixture of brewer’s yeast (Brewer’s Yeast, Healthy Life) and fish flakes (Wardley’s Tropical Fish Food Flakes, The Hartz Mountain Corporation, NJ), while 3rd and 4th instar larvae were fed on Hikari® Cichlid Staple pellets (Kyorin co. Ltd, Himeji, Japan). Pupae were placed in 150 mL containers within a 30 × 30 × 30 cm cage and emerged adults were provided 15% honey water *ad libitum*. Prior to virus exposure, mosquitoes were sorted by species and 5–7 day old females placed into 900 mL gauze-covered containers.

### Virus exposure

Mosquitoes were exposed for 2 hours to hanging drops of a blood/virus suspension containing stock virus, commercially available defibrinated sheep blood (Applied Biological Products Management – Australia, Aldinga Beach, Australia) and 1% sucrose [19]. For testing susceptibility to infection, *Cx. annulirostris* were offered blood meals containing 10-fold dilutions of stock virus, whilst *Cx. australicus* was exposed to a single dose of virus only. To determine the titer of virus at the time of feeding, 100 μL samples of the pre- and post-feeding blood/virus suspensions were diluted in 900 μL of growth media with antibiotics and antimycotics (GM; Gibco BRL®, Invitrogen, California) and supplemented with 3% fetal bovine serum (FBS), before being stored at −80°C.

Following feeding, mosquitoes were sorted and blood engorged mosquitoes placed in 900 mL gauze-covered containers. Containers were placed in sealed rigid plastic boxes within an environmental growth cabinet at 28°C and 12:12 L:D. Relative humidity was increased by placing a moist cotton wool pad within the plastic boxes and 10% sucrose was provided as a carbohydrate source.

### Measurement of susceptibility to infection, and virus dissemination and transmission

For the susceptibility trials, at day 14 post exposure, *Cx. annulirostris* fed the three lowest virus titers were killed via exposure to CO_2_ and placed individually in 2 mL vials containing 1 mL of GM +3% FBS and a 5 mm stainless steel ball. To compare virus replication and the length of the incubation period for the two viruses in *Cx. annulirostris*, on days 3, 5, 7, 10, 12 and 14, mosquitoes fed the highest virus titer were tested for their ability to transmit virus using the forced salivation method of Aitken [20]. Due to low survival rates following virus exposure, *Cx. australicus* were only tested for their ability to transmit at day 12. For transmission attempts, mosquitoes were anaesthetized with CO_2_ gas, their legs and wings removed and the proboscis of each mosquito inserted into a microcapillary tube containing 25 μL of GM +20% FBS. After approx. 30 min the contents of the tube were expelled into a 2 mL vial containing 500 μL of GM +3% FBS. The body remnants, and legs and wings were placed separately in 2 mL vials containing 1 mL of GM +3% FBS and a 5 mm stainless steel ball. Recovery of virus from the legs and wings is indicative of a disseminated infection, whereby the virus has escaped from the midgut and disseminated through the hemocoel [21]. The whole bodies, body remnants, and legs and wings, were homogenized in a QIAGEN TissueLyser II (Qiagen, Hilden, Germany) before being stored, along with the saliva expectorates, at −80°C.

### Virus detection

The pre- and post-exposure blood/virus suspensions were inoculated as 10-fold dilutions in the wells of a 96-well microtiter plate containing confluent layers of C6/36 cells. Plates were incubated at 28°C for seven days, after which time they were fixed with PBS/20% acetone with 0.2% BSA and stored at −20°C.

Mosquito homogenates were filtered through a 0.2 μm Supor® membrane filter (Pall Corporation, Ann Arbor, MI). The body filtrate of the mosquitoes exposed to the three lowest virus titers, and the legs and wings filtrate, were inoculated in duplicate onto C6/36 cell monolayers within a 96-well microtitre plate. The filtrate of the remaining bodies and all saliva expectorates were inoculated as 10-fold dilutions in the wells of a 96-well microtiter plate containing confluent monolayers of C6/36 cells. Plates were incubated, fixed and stored as described above.

A fixed cell culture enzyme immunoassay (CCEIA) was used to detect WNV_KUN_ infection in all blood/virus mixtures and mosquito samples using the WNV_KUN_- specific monoclonal antibody, 10A1 [22].

### Analysis

The titer of the blood/virus suspension, and the mosquito bodies and saliva expectorates was calculated using the method of Reed and Meunch [23] and expressed as TCID_50_/mL. The susceptibility of *Cx. annulirostris* to infection with the two WNV_KUN_ strains was calculated by probit analysis using PriProbit version 1.63 (Kyoto University, Kyoto, Japan). Log-log models were assessed using the Pearson chi-square goodness-of-fit statistic and susceptibility to infection was expressed as ID_50_ ± 95% confidence intervals (CIs) and defined as the virus dose per mL at which 50% of *Cx. annulirostris* tested positive for WNV_KUN_ infection in the CCEIA. Overlap of 95% CIs was used as a test of statistical significance.

Infection, dissemination and transmission rates in *Cx. annulirostris* and *Cx. australicus* exposed to WNV_KUN2011_ and WNV_KUN2009_ were compared using Fisher’s exact test [24]. Differences in virus titer within bodies and saliva expectorates from *Cx. annulirostris* and *Cx. australicus* exposed to WNV_KUN2011_ and WNV_KUN2009_ were analyzed for each day of exposure using *t*-tests [24].

## Results

### *Culex annulirostris* susceptibility to infection with WNV_KUN_

**Figure 1:**
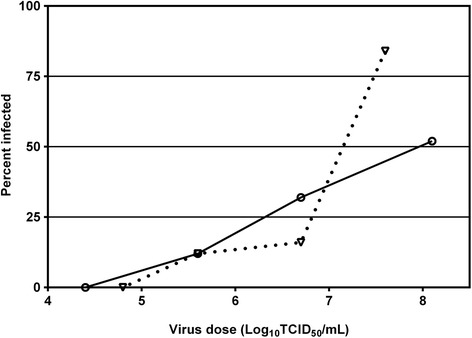
**Percent infection rates in** ***Culex annulirostris*** **exposed to serial dilutions of WNV**_**KUN2011**_ **(circles) and WNV**_**KUN2009**_ **(triangles) and tested at 14 d post-exposure.**

Mosquitoes were exposed to doses of WNV_KUN2011_ ranging from 10^4.4^ to10^8.1^ TCID_50_/mL, and 10^4.8^ to 10^7.6^ TCID_50_/mL for WNV_KUN2009_ (Figure 1). The susceptibilities of *Cx. annulirostris* to infection with the WNV_KUN2011_ and WNV_KUN2009_, expressed as ID_50_, were 10^7.9^ (10^7.4^-10^8.7^, 95% CI) TCID_50_ per mL (χ^2^ = 2.26, df = 2, *P* = 0.332) and 10^7.1^ (10^6.9^-10^7.4^, 95% CI) TCID_50_ per mL (χ^2^ = 5.58, df = 2 =, P = 0.06), respectively. Although there was no statistically significant difference in susceptibility to the virus strains, the overlap between CIs was very small.

### Infection and dissemination in and transmission by *Cx. annulirostris*

*Culex annulirostris* were exposed to virus titers of 10^8.1^ and 10^7.6^ TCID_50_/mL of WNV_KUN2011_ and WNV_KUN2009_, respectively. There was no significant difference (*P* > 0.05) in infection rates in *Cx. annulirostris* exposed to the two WNV_KUN_ strains on any day post exposure, except at day 14, where significantly more mosquitoes were infected with WNV_KUN2009_ compared to WNV_KUN2011_ (*P* = 0.0322; Table 1). There was no significant difference (*P* > 0.05) in the overall dissemination rate or the rate of dissemination in infected mosquitoes between *Cx. annulirostris* exposed to the two WNV_KUN_ strains on any day post-exposure (Table 1). Transmission for both virus strains was first observed at day 5 post-exposure, where 3/25 mosquitoes expectorated the virus (Table 1). There was no significant difference (*P* > 0.05) in the overall transmission rate or the transmission rate in mosquitoes with a disseminated infection on any day post-exposure.

**Table 1:** **Infection, dissemination and transmission rates in** ***Culex annulirostris*** **exposed to 10** ^**8.1**^ **and 10** ^**7.6**^ **TCID**_**50**_ **/mL of the WNV**_**KUN2011**_ **and WNV**_**KUN2009**_ **strains, respectively and tested at different time points post-exposure**

	**Infection** ^**a**^				**Dissemination** ^**b**^				**Dissemination/infection** ^**c**^				**Transmission** ^**d**^				**Transmission/dissemination** ^**e**^			
**Day post exposure**	**KUN2011**		**KUN2009**		**KUN2011**		**KUN2009**		**KUN2011**		**KUN2009**		**KUN2011**		**KUN2009**		**KUN2011**		**KUN2009**	
3	76	(19/25)	64	(16/25)	28	(7/25)	32	(8/25)	37	(7/19)	50	(8/16)	0	(0/25)	0	(0/25)	0	(0/7)	0	(0/8)
5	68	(17/25)	68	(17/25)	60	(15/25)	64	(16/25)	88	(15/17)	94	(16/17)	12	(3/25)	12	(3/25)	20	(3/15)	19	(3/16)
7	64	(16/25)	68	(17/25)	64	(16/25)	52	(13/25)	100	(16/16)	76	(13/17)	28	(7/25)	12	(3/25)	44	(7/16)	23	(3/13)
10	64	(16/25)	44	(11/25)	64	(16/25)	44	(11/25)	100	(16/16)	100	(11/11)	44	(11/25)	40	(10/25)	69	(11/16)	91	(10/11)
12	68	(17/25)	60	(9/15)	68	(17/25)	60	(9/15)	100	(17/17)	100	(9/9)	68	(17/25)	53	(8/15)	100	(17/17)	89	(8/9)
14	52	(13/25)	84	(21/25)*	52	(13/25)	76	(19/25)	100	(13/13)	90	(19/21)	52	(13/25)	72	(18/25)	100	(13/13)	95	(18/19)

^a^Percentage of mosquitoes containing virus in their bodies (number positive/number tested).

^b^Percentage of mosquitoes containing virus in their legs and wings (number positive/number tested).

^c^Percentage of infected mosquitoes containing virus in their legs and wings (number positive/number infected).

^d^Percentage of mosquitoes containing virus in the saliva expectorates (number positive/number tested).

^e^Percentage of mosquitoes with a disseminated infection containing virus in the saliva expectorates (number positive/number disseminated).

*Fisher’s exact test *P*-value <0.05 for day 14 infection rate.

**Figure 2:**
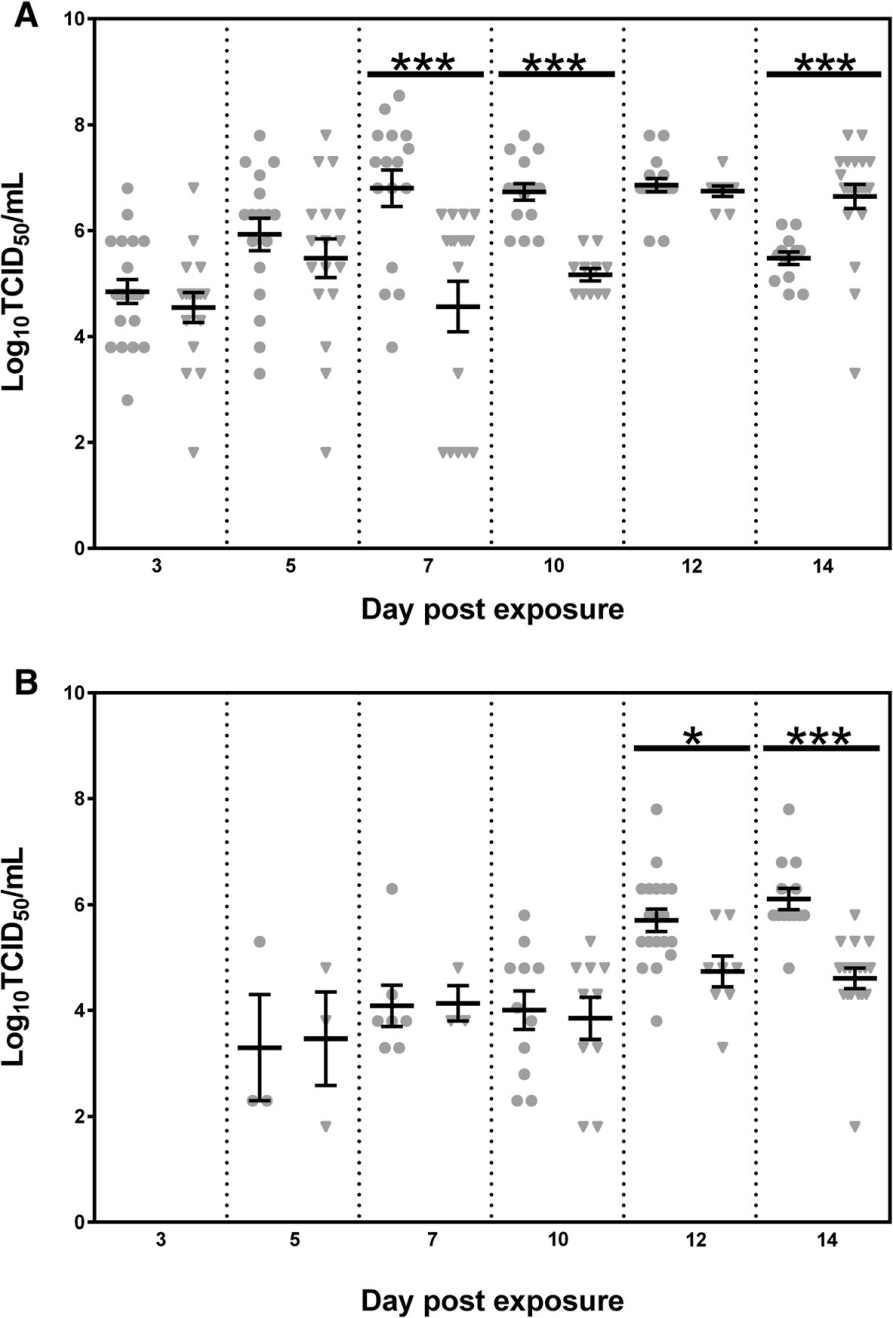
**Replication of WNV**_**KUN2011**_ **and WNV**_**KUN2009**_ **in the bodies (A) of** ***Culex annulirostris*** **and amount of virus expectorated in the saliva (B) by infected** ***Cx. annulirostris*** **, following the ingestion of an infectious blood meal.** Each point (circles for WNV_KUN2011_ and triangles for WNV_KUN2009_) represents an individual infected mosquito, and bars denote mean and standard error of the mean. *P* < 0.05 (*), *P* < 0.01 (**), *P* < 0.001 (***).

With the exception of day 14, body titers in *Cx. annulirostris* infected with WNV_KUN2011_ were higher than those infected with WNV_KUN2009_ (Figure 2A) and significantly higher on days 7 and 10 (*P* < 0.001). Conversely, on day 14, WNV_KUN2009_-infected mosquitoes had significantly higher body titers than WNV_KUN2011_-infected mosquitoes (*P* < 0.001), which coincided with the significantly higher infection rate observed above. On days 10–14, when a greater proportion of mosquitoes was transmitting the virus, there was a higher amount of virus expectorated by WNV_KUN2011_-infected mosquitoes, with significant differences observed on days 12 and 14 (*P* < 0.05; Figure 2B).

### Infection and dissemination in and transmission by *Cx. australicus*

There was no significant difference (*P* > 0.05) in infection, dissemination and transmission rates in *Cx. australicus* 12 days after being exposed to virus titers of 10^8.1^ and 10^7.6^ TCID_50_/mL of WNV_KUN2011_ and WNV_KUN2009_, respectively (Table 2). The infection and dissemination rates were also not significantly different (*P* > 0.05) from those observed for *Cx. annulirostris*. However, the overall transmission rate was significantly lower (*P* < 0.05) in *Cx. australicus* than *Cx. annulirostris* for both WNV_KUN2011_ and WNV_KUN2009_ (Table 2). Similarly, the transmission rate in mosquitoes with a disseminated infection was lower in *Cx. australicus* than *Cx. annulirostris*, with the difference being significant (*P* < 0.05) for WNV_KUN2011_.

**Table 2:** **Infection, dissemination and transmission rates in** ***Culex australicus*** **and** ***Culex annulirostris*** **exposed to 10** ^**8.1**^ **and 10** ^**7.6**^ **TCID**_**50**_ **/mL of the WNV**_**KUN2011**_ **and WNV**_**KUN2009**_ **strains, respectively, and tested at day 12 post-exposure**

	**Infection** ^**a**^				**Dissemination** ^**b**^				**Dissemination/infection** ^**c**^				**Transmission** ^**d**^				**Transmission/dissemination** ^**e**^			
**Species**	**KUN2011**		**KUN2009**		**KUN2011**		**KUN2009**		**KUN2011**		**KUN2009**		**KUN2011**		**KUN2009**		**KUN2011**		**KUN2009**	
*Cx. australicus*	68	(17/25)	50	(11/22)	60	(15/25)	41	(9/22)	88	(15/17)	82	(9/11)	20	(5/25)*	18	(4/22)*	33	(5/15)*	44	(4/9)
*Cx. annulirostris*	68	(17/25)	60	(9/15)	68	(17/25)	60	(9/15)	100	(17/17)	100	(9/9)	68	(17/25)	53	(8/15)	100	(17/17)	89	(8/9)

^a^Percentage of mosquitoes containing virus in their bodies (number positive/number tested).

^b^Percentage of mosquitoes containing virus in their legs and wings (number positive/number tested).

^c^Percentage of infected mosquitoes containing virus in their legs and wings (number positive/number infected).

^d^Percentage of mosquitoes containing virus in the saliva expectorates (number positive/number tested).

^e^Percentage of mosquitoes with a disseminated infection containing virus in the saliva expectorates (number positive/number disseminated).

*Fisher’s exact test *P*-value <0.05 for comparisons between *Cx. australicus* and *Cx. annulirostris*.

**Figure 3:**
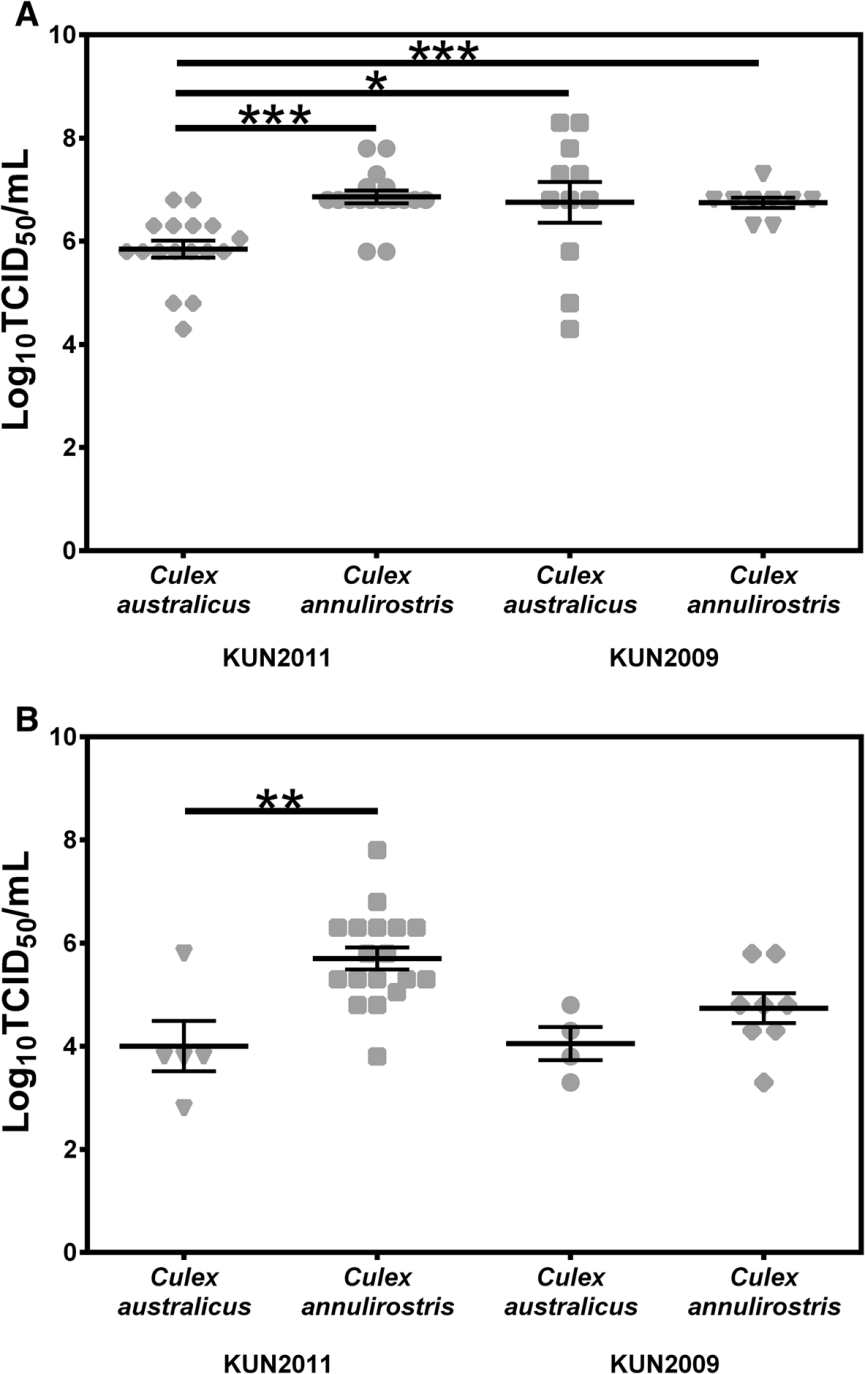
**Replication of WNV**_**KUN2011**_ **and WNV**_**KUN2009**_ **in the bodies (A) of** ***Culex australicus*** ***and*** ***Culex annulirostris*** **and amount of virus expectorated in the saliva (B) by infected** ***Cx. australicus*** **and** ***Cx. annulirostris*** **, 12 days after ingesting an infectious blood meal.** Each point on the plot represents an individual infected mosquito, and bars denote mean and standard error of the mean. *P* < 0.05 (*), *P* < 0.01 (**), *P* < 0.001 (***).

The body titers in *Cx. australicus* exposed to WNV_KUN2011_ were significantly lower (*P* < 0.05) than those exposed to WNV_2009_ (Figure 3A). They were also significantly lower (*P* < 0.001) than the body titers observed for both viruses in *Cx. annulirostris*. There was no significant difference (*P* > 0.05) between the amount of WNV_KUN2011_ or WNV_KUN2009_ expectorated by *Cx. australicus* (Figure 3B). However, lower amounts of each virus strain were expectorated by *Cx. australicus* when compared to *Cx. annulirostris*, with this difference significant (*P* < 0.05) for WNV_KUN2011_.

## Discussion

Following the 2011 epidemic, key genetic, antigenic and phenotypic characteristics of WNV_KUN2011_ were investigated to ascertain why there was such a dramatic impact from what was considered to be a relatively attenuated strain of WNV [6]. In mouse virulence studies, WNV_KUN2011_ was considerably more neuroinvasive than the prototype WNV_KUN_ and WNV_KUN2009_ in both weanling and young adult mice ([6]; Prow et al. unpublished data).

The report by Frost et al. [6] suggested that the WNV_KUN2011_ was more virulent and may be transmitted more efficiently between mosquitoes and mammalian hosts. In the current study, we assessed a number of attributes that constitute the vector competence of a mosquito species, including susceptibility to infection, virus replication dynamics and ability to transmit the virus. Although there was no significant difference in transmission rate of the two viruses by *Cx. annulirostris*, more WNV_KUN2011_ was expectorated than WNV_KUN2009_ on days 10–14 post-exposure and significantly more on days 12 and 14. Given the high titers of virus that mosquitoes were exposed to in the transmission experiments, we believe that the 10^0.5^ TCID_50_/mL difference in titer between the WNV_KUN2011_ and WNV_KUN2009_ blood meals would have had a negligible impact on the outcomes of this study.

An increased amount of virus expectorated by mosquitoes may lead to hosts being inoculated with a higher dose of virus than would occur with other WNV_KUN_ strains. However, we are unaware of any study that provides the precise dose of virus expectorated by a mosquito that is required to infect a vertebrate host or cause clinical disease in a host. Therefore, needle inoculation is often used as a proxy for mosquito infection and to standardize the dose of virus inoculated into a host. Using this mode of infection, it has been shown that higher doses lead to earlier and more enhanced WNV viremia levels [25,26] and increased oral shedding [27] in birds than lower doses. Thus, it is plausible that during the 2011 epidemic, infection of ardeid birds by higher doses of WNV_KUN2011_ may have led to a more rapid onset of an elevated viremia, which, in turn, led to the infection of a greater proportion of mosquitoes, a higher rate of virus replication and increased transmission. Furthermore, higher amounts of virus expectorated by mosquitoes may have led to a greater proportion of horses being inoculated with a dose of virus capable of inducing clinical disease. A dose response to infection has been demonstrated with a number of WNV strains in mouse models, with higher doses leading to more rapid onset of disease and higher mortality [28,29].

In addition to increased transmissibility of WNV_KUN2011_ by mosquitoes, other intrinsic and extrinsic factors likely influence the role of *Cx. annulirostris* in transmission cycles. Some of these factors are important parameters that are used to assess the vectorial capacity of a given species for an arbovirus and include daily survival rate, extrinsic incubation period (EIP) of the virus, population density, and host feeding behaviour [30]. Because an infected mosquito must survive the EIP for transmission to occur, mosquito survival is one of the key components of vectorial capacity. Field studies estimate the daily survival rate of *Cx. annulirostris* to be 70-85%, so it has been estimated that < 20% of the population survive the 8–10 day EIP to transmit MVEV, another important encephalitic flavivirus in Australia [31-33]. Considering that it was at days 12 and 14 post-exposure when we significantly higher amounts of WNV_KUN2011_ being expectorated, it is likely that only a very small proportion of the *Cx. annulirostris* population would survive to transmit at these time points in the field, potentially offsetting the field impact of our laboratory observation.

The significantly higher than average rainfall experienced during 2010 and 2011 produced widespread freshwater larval habitats suitable for *Cx. annulirostris*. Thus, it was not surprising that there was an explosive increase in density of this species, with *Cx. annulirostris* dominant in trap collections that yielded over 10,000 mosquitoes per trap [12]. Widespread flooding would also have provided an ideal habitat for ardeid birds [4]. Analysis of host feeding patterns has previously identified *Cx. annulirostris* blood meals from the Rufous Night Heron, *Nycticorax caledonicus* [34], so the convergence between high populations of amplifying hosts and vectors may have contributed to the widespread nature of the outbreak. Given that *Cx. annulirostris* is an opportunistic blood feeder [34], this species may have not only acted as an epizootic vector cycling the virus between birds, but may have served as a bridge vector transmitting the virus to susceptible horses.

In our experiments, *Cx. australicus* did not have increased vector competence for WNV_KUN2011_ compared to WNV_KUN2009_ and had a significantly lower transmission rate for both virus strains than *Cx. annulirostris*. Although no isolates of WNV_KUN2011_ were obtained from *Cx. australicus* during the 2011 outbreak, this species has yielded WNV_KUN_ isolates previously [35]. Despite this, given that *Cx. australicus* feeds predominately on birds [36,37] it may have played a role in epizootic amplification of the virus, especially where populations were higher than average. The substantial role of poorly competent vectors in arbovirus transmission cycles has been demonstrated previously, with biological traits, such as specific host feeding patterns or high population densities negating relatively low transmission rates [38,39].

Although the primary objective of the current study was to characterize WNV_KUN2011_ in *Cx. annulirostris*, it is possible that other mosquito species may have contributed to the 2011 epidemic. Another member of the *Cx. pipiens* group, *Culex quinquefasciatus* is a highly efficient vector of WNV_NY99_ [40], exhibits ornithophilic host feeding patterns [34] and was abundant at some sites during the WNV_KUN2011_ outbreak [12]. While there is no data on local vector competence of *Culex molestus*, this species, as part of the *Cx.pipiens* group globally, is widely distributed within the region of WNV_KUN_ activity [41] and based on international studies, should be considered a potential vector [42]. Finally, floodwater *Aedes*, such as *Aedes theobaldi*, *Aedes vittiger*, *Aedes eidsvoldensis* and *Aedes sagax* were also abundant during the 2010–2011 season [12] and may have played a role in transmission. Clearly, further studies are required to elucidate the role of other mosquito species in transmission of WNV_KUN2011_ and related flaviviruses.

The 2011 outbreak of equine encephalitis attributed to infection with WNV_KUN2011_ was unprecedented. It was geographically widespread and WNV_KUN_ activity appeared for the first time near populous centers of the east coast. Above average rainfall again triggered high mosquito population densities in some inland locations during the 2011–2012 season [43]. However, it did not result in continued widespread virus activity and very few cases of neurological disease in horses have been reported since 2010–2011 [44]. A 2012 WNV_KUN_ isolate showed similar levels of virulence asWNV_KUN2011_ in mice (Prow et al. unpublished data), suggesting continued circulation of this pathogenic strain.

## Conclusions

To assess the relative fitness of WNV_KUN2011_ in *Cx. annulirostris*, we examined susceptibility of this species to infection, replication dynamics and ability to transmit the virus. One of the key findings of the study was more efficient transmission of this virus strain at the later days post infection, when compared to the less pathogenic WNV_KUNV2009_ strain. Because WNV transmission cycles involve a complex interaction between the virus, vertebrate host and mosquito vector [1], results from laboratory-based experiments should not be viewed in isolation but need to be placed into an ecological context. Therefore it is more likely that a combination of more efficient transmission of WNV_KUN2011_ by *Cx. annulirostris*, elevated mosquito populations, an abundance of ardeid birds serving as amplifying hosts, opportunistic blood feeding habits of the key vector and increased pathogenicity of the virus in horses may have driven the 2011 equine epidemic in southeastern Australia.
